# Association between frailty and depression in older adults with coronary heart disease: a systematic review and meta-analysis

**DOI:** 10.3389/fpubh.2026.1737823

**Published:** 2026-04-13

**Authors:** Yuan Chen, Zhaoxia Zhang, Yuanling Tang, Shixia Zhang, Yaling Huang, Wenting Chai, Qixi Liu

**Affiliations:** 1 Department of Nursing, Fujian University of Traditional Chinese Medicine, Fuzhou, Fujian, China; 2 Cardiovascular Hospital of Xiamen University, Xiamen, Fujian, China; 3 The First Affiliated Hospital of Xiamen University, Xiamen, Fujian, China; 4 Mindong Hospital of Ningde Affiliated to Fujian Medical University, Ningde, Fujian, China

**Keywords:** association, coronary heart disease, depression, frailty, meta-analysis

## Abstract

**Background:**

Frailty and depression frequently co-occur in older patients with coronary heart disease (CHD) and are each associated with adverse outcomes. However, the strength and nature of their association in this population have not been systematically quantified.

**Methods:**

We systematically searched PubMed, Embase, and Web of Science and other major databases from inception to April 2025 for observational studies examining the association between frailty and depression in adults with CHD. Two reviewers independently screened studies, extracted data, and assessed quality using the Newcastle-Ottawa Scale for cohort studies and the AHRQ checklist for cross-sectional studies. Both cross-sectional and cohort studies were eligible. Pooled odds ratios (ORs) with 95% confidence intervals (CIs) were calculated using a random-effects model, prioritizing adjusted estimates where available. Heterogeneity was quantified using the *I^2^* statistic. Subgroup analyses were planned by region, study design and assessment tool.

**Results:**

Of 2,160 records screened, 6 studies met the inclusion criteria, comprising 2,628 patients with CHD. All included studies focused on older adults (mean/median age ≥60 years), reflecting the current evidence base in this population. Meta-analysis showed a significant positive association between frailty and depression (pooled OR = 2.45; 95% CI: 1.22–4.92), with substantial heterogeneity (*I^2^* = 90%). When analyzed by study design, the association appeared more pronounced in cohort studies (OR = 3.92; 95% CI: 1.66–9.27; *I^2^* = 71%) than in cross-sectional studies (OR = 1.91; 95% CI: 1.02–3.58; *I^2^* = 74%).

**Conclusion:**

Frailty and depression are significantly associated in older adults with CHD, supporting integrated screening approaches. However, given the predominance of cross-sectional data, high heterogeneity, and limited cohort evidence, prospective studies are needed to establish temporality and better inform causal inference.

**Systematic review registration:**

https://www.crd.york.ac.uk/PROSPERO/view/CRD420251036929, PROSPERO (CRD420Ò51036929).

## 1 Introduction

Coronary heart disease (CHD) represents a leading cause of global mortality, accounting for an estimated 7.4 million deaths annually (1, 2). Beyond mortality, CHD profoundly impairs patients’ quality of life and imposes a substantial economic burden (3, 4). Established risk factors contributing to CHD incidence include diabetes, smoking, dyslipidemia, and hypertension (5–8). Recently, increasing attention has been given to patient-centered vulnerability factors, particularly frailty and depression, which are not only common in CHD patients but also independently associated with worse prognosis (9–11).

Frailty is a progressive geriatric syndrome resulting from diminished physiological reserves. It is primarily characterized by reduced muscle strength and physiological function, increasing an individual’s vulnerability to stressors and mortality risk (12). The prevalence of frailty in CHD patients varies considerably, ranging up to 60% depending on the population studied and assessment method used (13–15). For instance, Damluji et al. (13) reported that CHD patients are approximately three times more likely to develop frailty than those without CHD. Frailty in this population predisposes individuals to prolonged hospitalization, disability, postoperative complications, and mortality, collectively diminishing quality of life (16, 17).

Depression, a common mental disorder, constitutes a major contributor to the global disease burden and is a leading cause of disability worldwide (18). Evidence from multiple studies indicates that depression prevalence among CHD patients ranges from 20 to 50% (19), substantially exceeding rates in the general population. Furthermore, depression increases the risk of recurrent myocardial infarction by approximately 30% and is linked to a 1.8- to 2-fold increase in mortality (20).

Emerging evidence suggests that frailty and depression frequently co-occur in patients with CHD, and their coexistence may have synergistic adverse effects on clinical outcomes. Research in older populations indicates that frailty, characterized by declines in somatic function, can lead to disability or functional dependence. This functional decline may subsequently induce or exacerbate depressive symptoms (21).

Conversely, depression-related behavioral changes such as physical inactivity, poor medication adherence, and social withdrawal may accelerate functional decline and frailty progression (22, 23). Such manifestations of depression can further intensify core affective symptoms, including persistent sadness and helplessness. This complex interplay may create a potential vicious cycle that could disproportionately worsen prognosis in CHD populations compared to general older adults (24). Given the high co-occurrence of depression and frailty among CHD patients and the amplified adverse outcomes associated with their coexistence, elucidating the association between these two conditions is imperative.

Although several studies have examined the association between frailty and depression in patients with CHD, their findings have been inconsistent, likely due to differences in study populations, frailty assessment tools, and depression measures. To date, no systematic review or meta-analysis has quantitatively synthesized this evidence specifically in CHD populations. This gap limits the development of targeted screening strategies and integrated care models for high-risk patients. Therefore, this systematic review and meta-analysis aims to: (1) quantitatively synthesize the existing evidence on the association between frailty and depression in patients with CHD; (2) explore potential sources of heterogeneity through subgroup analyses based on study design, region, and assessment tools; and (3) provide an evidence base to inform clinical practice and guide future research on integrated management of frailty and depression in CHD populations.

## 2 Materials and methods

This systematic review and meta-analysis was conducted and reported following the guidelines of the Preferred Reporting Items for Systematic Reviews and Meta-Analyses (PRISMA) (25) and the Meta-analysis of Observational Studies in Epidemiology (MOOSE) (26). The study protocol was registered in the International Prospective Register of Systematic Reviews (PROSPERO; ID: CRD420251036929).

### 2.1 Search strategy

A comprehensive literature search was performed across seven electronic databases, including PubMed, Embase, Web of Science, Cochrane Library, CINAHL, Scopus, and relevant gray literature sources, from their inception to April 12, 2025. The search strategy combined terms related to coronary heart disease (e.g., “coronary artery disease,” “myocardial infarction”), frailty (e.g., “frailty,” “frailty syndrome”), and depression (e.g., “depression,” “depressive disorder”), using Boolean operators (AND, OR, NOT). The full search strategies for each database are provided in Supplementary Table 1.

### 2.2 Eligibility criteria

Inclusion Criteria: (1) Participants: Studies that included adult participants diagnosed with CHD; (2) Exposure/Outcome Association: Studies that reported on the association between frailty and depression among the CHD participants, assessed using validated measurement tools; (3) Study Design: Observational studies with cross-sectional or cohort designs were eligible for inclusion.

Exclusion Criteria: (1) Duplicate publications, or studies presenting incomplete, unclear, or evidently erroneous data; (2) Lectures, conference abstracts (without full peer-reviewed papers), commentaries, editorials, case reports, or animal studies; (3) Articles for which the full text could not be obtained despite contacting the first or corresponding author via email; (4) Studies that lacked sufficient data for quantitative synthesis (e.g., could not provide or allow calculation of odds ratios with confidence intervals); (5) Studies reporting only continuous depression scores without providing a validated categorical diagnosis of depression (e.g., based on established diagnostic criteria or validated cut-off scores).

### 2.3 Study selection and data extraction

Two researchers (CY and ZZX) independently conducted the study selection. Disagreements were resolved through discussion or, when necessary, by consulting a third reviewer (TYL), an expert in evidence synthesis. Initially, duplicate records were identified and removed using EndNote 21 software. Subsequently, the same researchers screened the titles and abstracts of the remaining records against the predefined inclusion and exclusion criteria. A standardized data extraction form was developed in Microsoft Excel. Two researchers (CY and ZZX) independently extracted the following data from each eligible study: (1) study characteristics, including first author, publication year, study location (country/region), and study design; (2) participant characteristics, such as sample size, age, and gender distribution; (3) frailty measures, including the assessment tool used and the reported prevalence of frailty; (4) depression measures, including the assessment tool used and the reported prevalence of depression; and (5) association metrics, specifically odds ratios (ORs) with 95% confidence intervals (CIs). Discrepancies in data extraction were resolved through consensus discussion or by involving the third researcher (TYL).

### 2.4 Assessment of study quality

The methodological quality of the included studies was assessed independently by two reviewers (CY and ZZX). For cross-sectional studies, quality was evaluated using the 11-item checklist developed by the Agency for Healthcare Research and Quality (AHRQ) (27). Based on the total score, studies were categorized as follows: low quality (0–3 points), moderate quality (4–7 points), or high quality (8–11 points). For case–control and cohort studies, quality was assessed using the Newcastle-Ottawa Scale (NOS) (28), in accordance with Cochrane Collaboration recommendations. The NOS evaluates studies across three domains using eight items, with a maximum score of 9 points. Studies were classified as low quality (≤4 points), moderate quality (5–6 points), or high quality (≥7 points). Any discrepancies between the reviewers’ quality assessments were resolved through discussion or, if necessary, by consulting a third reviewer (TYL). The detailed quality scores and ratings for each included study are provided in Tables 1, 2.

**Table 1:** Methodological quality appraisal results based on the AHRQ tool for each study.

Study	Study design	Item 1	Item 2	Item 3	Item 4	Item 5	Item 6	Item 7	Item 8	Item 9	Item 10	Item 11	Total score	Quality
Uchmanowicz et al. (46)	Cross-sectional	Y	Y	Y	N	N	Y	Y	Y	N	Y	N	7	M
Aggarwal et al. (47)	Cross-sectional	Y	Y	Y	Y	N	Y	Y	Y	N	Y	N	8	H
Dai et al. (48)	Cross-sectional	Y	Y	Y	Y	N	Y	Y	Y	N	Y	N	8	H
Zakiev et al. (49)	Cross-sectional	Y	Y	Y	Y	N	Y	Y	Y	N	Y	N	8	H

Y, yes; N, no; U, unclear; H, high quality; M, medium quality. Item 1: Define the source of information (survey, record review). Item 2: List inclusion and exclusion criteria for exposed and unexposed subjects (cases and controls) or refer to previous publications. Item 3: Indicate time period used for identifying patients. Item 4: Indicate whether or not subjects were consecutive if not population-based. Item 5: Indicate if evaluators of subjective components of the study were masked to other aspects of the status of the participants. Item 6: Describe any assessments undertaken for quality assurance purposes (e.g., test/retest of primary outcome measurements). Item 7: Explain any patient exclusions from analysis. Item 8: Describe how confounding was assessed and/or controlled. Item 9: If applicable, explain how missing data were handled in the analysis. Item 10: Summarize patient response rates and completeness of data collection. Item 11: Clarify what follow-up, if any, was expected and the percentage of patients for which incomplete data or follow-up was obtained.

**Table 2:** Methodological quality appraisal results based on the NOS tool for each study.

Study	Study design	Selection				Comparability	Outcome			Total score	Quality
		Item 1	Item 2	Item 3	Item 4	Item 5	Item 6	Item 7	Item 8		
Kang et al. (45)	Cohort	1	1	0	0	2	1	0	1	6	M
Desai et al. (29)	Cohort	1	0	1	0	2	1	0	0	5	M
Damluji et al. (13)	Cohort	1	1	1	1	2	1	1	1	9	H

H, high quality; M, medium quality. Item 1: Representativeness of the exposed cohort. Item 2: Selection of the non-exposed cohort. Item 3: Ascertainment of exposure. Item 4: Demonstration that the outcome of interest was not present at the start of the study. Item 5: Comparability of cohorts on the basis of the design or analysis. Item 6: Assessment of outcome. Item 7: Was the follow-up long enough for outcomes to occur. Item 8: Adequacy of follow-up of cohorts.

### 2.5 Statistical analysis

All meta-analyses were conducted using R software (version 4.4.3). The primary measure of association was the pooled odds ratio (OR) with its corresponding 95% confidence interval (CI). Of note, risk ratios would theoretically be more appropriate given the high prevalence of both conditions in CHD populations, but these were not reported in the primary studies. Where available, we prioritized adjusted ORs that accounted for potential confounders. However, due to inconsistent reporting and the limited number of studies providing adjusted estimates, crude (unadjusted) ORs were extracted from the majority of included studies. For studies that reported both crude and adjusted estimates, the adjusted values were used when available. Effect estimates were combined using the inverse variance method. Heterogeneity was assessed using Cochran’s *Q* test and quantified by the *I^2^* statistic. Substantial heterogeneity was defined as *I^2^* ≥ 50% and/or a *Q*-test *p* ≤ 0.10. When heterogeneity was not substantial (*I^2^* < 50% and *p* > 0.10), a fixed-effects model was applied. Otherwise, a random-effects model was used. Pre-specified subgroup analyses were performed to explore potential sources of heterogeneity based on study design, geographic region, frailty assessment tools, and depression assessment tools. Sensitivity analyses were conducted to evaluate the robustness of the pooled results by examining the influence of individual studies and the choice of effect model. Publication bias was evaluated using funnel plots, Begg and Mazumdar’s rank correlation test, and Egger’s linear regression test. Evidence of significant publication bias was considered when *p* ≤ 0.05 for the Begg or Egger tests. Conversely, a *p* > 0.05 was interpreted as indicating no significant evidence of publication bias.

## 3 Results

### 3.1 Search results summary

**Figure 1:**
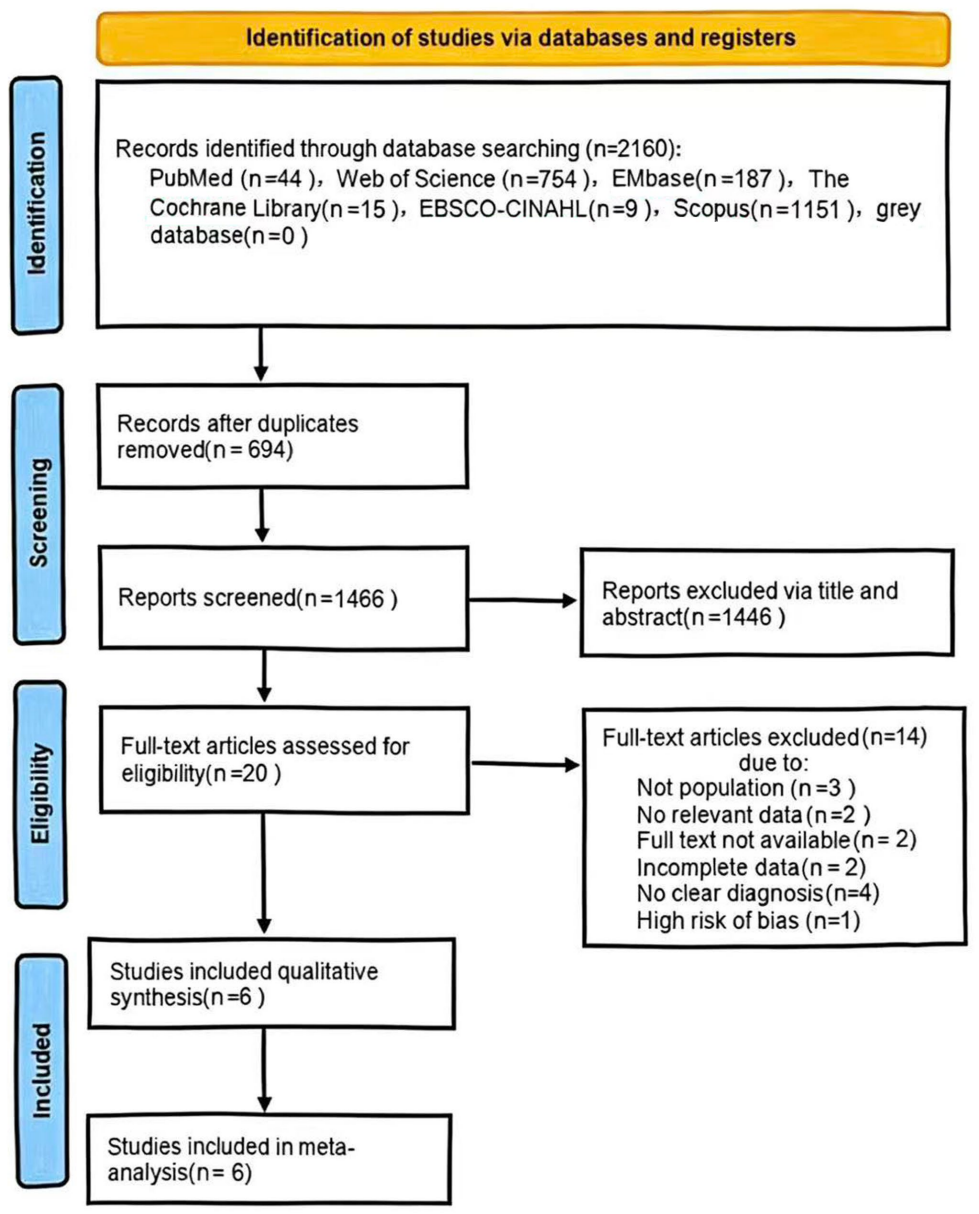
PRISMA flowchart showing the selection process of observational studies for inclusion in the meta-analysis.

The initial electronic database search identified 2,160 records. After removing 694 duplicates, 1,466 unique records remained. Screening of titles and abstracts excluded 1,446 records as irrelevant. Full-text assessment was performed for the remaining 20 articles. Following application of the eligibility criteria to the full texts, seven studies were provisionally selected for inclusion. The methodological quality of these seven studies was then evaluated. One study was subsequently excluded due to a quality score below the pre-defined threshold (NOS score <6). Consequently, six studies were included in the final systematic review and meta-analysis. The study selection process, detailing the number of records at each stage, is presented in the PRISMA flow diagram (Figure 1).

### 3.2 Characteristics of the included studies

Although our search strategy and eligibility criteria did not restrict inclusion by age, all six studies that met the inclusion criteria were conducted in older adult populations with CHD (defined as mean or median participant age ≥60 years). The mean or median age exceeded 60 years in every study. The six included studies encompassed a total of 2,628 patients with CHD. Key characteristics are summarized in Table 3. Publication years ranged from 2015 to 2024. Study designs comprised four cross-sectional and two cohort studies. Geographically, four studies originated in Asia, one in the United States, and one in Europe. In terms of assessment tools, frailty was assessed using the Fried phenotype in three studies. Depression was measured using the Hospital Anxiety and Depression Scale (HADS) in two studies and the 15-item Geriatric Depression Scale (GDS-15) in another two. The remaining studies employed other validated instruments for frailty and depression assessment.

**Table 3:** Characteristics of the included studies.

Author	Country	Study design	Sample size (n)	Age range (years)	Percentage of male (%)	Percentage of female (%)	Type of disease	Frailty criterion	Depression criterion	Prevalence of frailty (%)	Prevalence of depression (%)	OR (95% CI)
Kang et al. (45)	China	Cohort	352	≥65	57.70	42.30	ACS	CFS	HADS	43.18	12.55	2.24(0.91,5.56)
Uchmanowicz et al. (46)	Poland	Cross-sectional	135	69.8 ± 11.4	60.70	39.30	ACS	TFI	HADS	77.80	67.40	6.75(2.79,16.36)
Aggarwal et al. (47)	India	Cross-sectional	100	≥60	73.00	27.00	ACS	FP	GDS	44.00	40.00	1.36(0.55,3.39)
Damluji et al. (13)	America	Cohort	1,213	79.8	49.50	50.50	CHD	FP	PHQ-2	28.60	21.40	5.54(4.13,7.42)
Dai et al. (48)	China	Cross-sectional	288	≥60	60.10	39.90	CCS	FP	GDS-15	37.50	49.00	1.46(0.82,2.58)
Zakiev et al. (49)	Russia	Cross-sectional	540	78.3 ± 8.4	59.80	40.20	MI	CGA	GDS-15	70.20	57.90	1.30(0.90,1.89)

ACS, Acute Coronary Syndrome; CHD, Coronary Heart Disease; CCS, Chronic Coronary Syndrome; MI, Myocardial Infarction; FP, Fried Frailty Phenotype; CFS, Clinical frailty scale; TFI, Tilburg frailty indicator; CGA, Comprehensive Geriatric Assessment; GDS, Geriatric Depression Scale; PHQ-2, Patient Health Questionnaire-2; HADS, Hospital Anxiety and Depression Scale; GDS-15, Geriatric Depression Scale-15.

### 3.3 Quality assessment

The methodological quality of the included studies was assessed using the NOS for cohort studies and the AHRQ assessment criteria for cross-sectional studies. Cohort studies (assessed with NOS): Scores ranged from 5 to 9. The study that scored 5 was excluded due to its low methodological quality, in order to minimize potential risk of bias. Consequently, two cohort studies with NOS scores of 6 and 9 were retained. Cross-sectional studies (assessed with AHRQ): Scores ranged from 7 to 8. All four cross-sectional studies met the threshold for moderate to high quality and were included. Detailed quality scores and ratings for each study are provided in Tables 1, 2.

### 3.4 Association between frailty and depression

**Figure 2:**
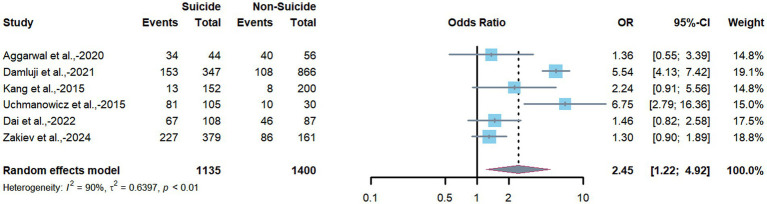
Forest plot of pooled the association between frailty and depression in CHD patients.

The pooled association between frailty and depression, synthesized using a random-effects model based on data from the six included studies, demonstrated a statistically significant positive association (pooled OR = 2.45; 95% CI = 1.22–4.92; *p* = 0.002). Heterogeneity among the included studies was substantial (*I^2^* = 90%). The forest plot depicting individual study estimates and the pooled result is presented in Figure 2.

### 3.5 Subgroup analysis

To explore potential sources of heterogeneity, we performed subgroup analyses based on study design, geographic region, frailty assessment tool, and depression assessment tool. When analyzed by study design, a significant association between frailty and depression was observed in both study types. The pooled effect size was larger among the two cohort studies (OR = 3.92; 95% CI: 1.66–9.27; *I^2^* = 71%; based on 2 studies, 1,565 participants) than among the four cross-sectional studies (OR = 1.91; 95% CI: 1.02–3.58; *I^2^* = 74%; based on 4 studies, 1,063 participants). In the subgroup analysis by geographic region, a significant association was observed in all regions: Asia (OR = 1.42; 95% CI: 1.07–1.87; based on 4 studies, 1,280 participants), Europe (OR = 5.54; 95% CI: 4.13–7.42; based on 1 study, 135 participants), and America (OR = 6.75; 95% CI: 2.79–16.36; based on 1 study, 1,213 participants).

In subgroup analyses by frailty assessment tool, a significant association was observed only in studies using the TFI (OR = 6.75, 95% CI: 2.79–16.36; based on 1 study, 135 participants). No significant associations were observed in studies using the FP (OR = 2.33; 95% CI: 0.80–6.73; based on 3 studies, 1,601 participants), the CFS (OR = 2.24; 95% CI: 0.91–5.56; based on 1 study, 352 participants), or the CGA (OR = 1.30; 95% CI: 0.90–1.89; based on 1 study, 540 participants).

For depression assessment tools, significant associations emerged in studies using the HADS (OR = 3.91, 95% CI: 1.33–11.51; based on 2 studies, 487 participants) and the PHQ-2 (OR = 5.54, 95% CI: 4.13–7.42; based on 1 study, 1,213 participants). In contrast, no significant associations were observed in studies using the GDS-15 (OR = 1.35; 95% CI: 0.99–1.84; based on 2 studies, 828 participants) or the GDS (OR = 1.36; 95% CI: 0.55–3.39; based on 1 study, 100 participants). These results are summarized in Table 4.

**Table 4:** Subgroup analyses stratified by region, frailty instrument, and depression instrument.

Subgroup	Number of studies	Effect model	Meta-analysis		Heterogeneity	
			OR	95%CI	*I^2^*	*P*
Region						
Asia	4	Fixed	1.42	1.07–1.87	0	0.75
America	1	Fixed	5.54	4.13–7.42	–	–
Europe	1	Fixed	6.75	2.79–16.36	–	–
Frailty instrument						
FP	3	Random	2.33	0.80–6.73	91	<0.001
CFS	1	Random	2.24	0.91–5.56	–	–
TFI	1	Random	6.75	2.79–16.36	–	–
CGA	1	Random	1.3	0.90–1.89	–	–
Depression instrument						
GDS-15	2	Fixed	1.35	0.99–1.84	0	0.09
HADS	2	Random	3.91	1.33–11.51	66	0.09
GDS	1	Random	1.36	0.55–3.39	–	–
PHQ-2	1	Random	5.54	4.13–7.42	–	–
Study design						
Cross-sectional	4	Random	1.91	1.02–3.58	74	0.009
Cohort	2	Random	3.92	1.66–9.27	71	0.06

CI, confidence interval; OR, odds ratio.

### 3.6 Sensitivity analysis and study exclusion rationale

Substantial heterogeneity was observed (*I^2^* = 90%) during meta-analysis. To evaluate result robustness and investigate potential sources of heterogeneity, sensitivity analyses were performed by iteratively excluding individual studies. These analyses were based on all seven studies that initially met the inclusion criteria prior to quality assessment. One study by Desai et al. (29) reported an inverse association (OR < 1), in contrast to the positive associations observed in other studies. This study also scored below the pre-defined quality threshold (NOS score = 5). Based on our protocol, studies with NOS scores below 6 were considered low quality and were excluded to maintain methodological rigor. Therefore, Desai et al. (29) was excluded from the final meta-analysis.

**Figure 3:**
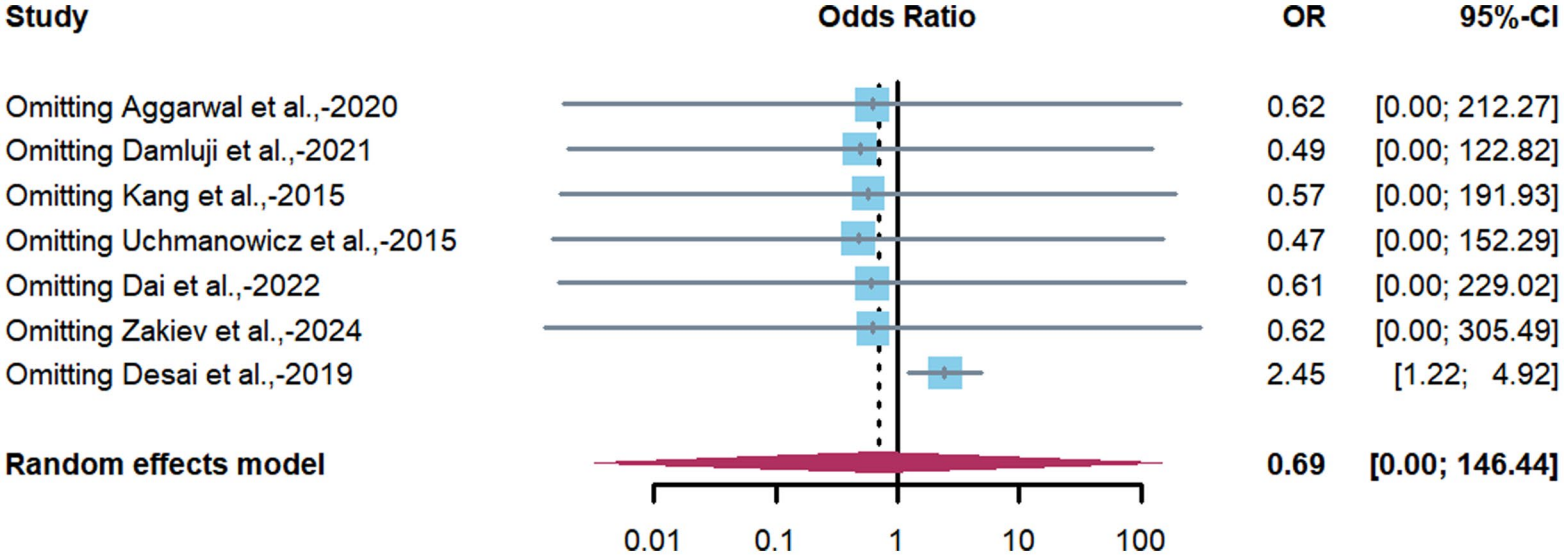
Forest plot of sensitivity analysis of the association between frailty and depression in CHD patients. Forest plot showing the influence of individual studies on the pooled estimate. This analysis includes all seven studies that initially met the inclusion criteria, prior to the exclusion of one low-quality study (29). The pooled estimates represent the results after iteratively removing each study.

After excluding this study, the direction and significance of the pooled estimate remained consistent, supporting the robustness of the overall finding. However, the exclusion did not substantially reduce the overall heterogeneity (*I^2^* remained at 90%), indicating that this single study was not the primary source of variability. The forest plot of sensitivity analysis is presented in Figure 3.

### 3.7 Heterogeneity and publication bias

**Figure 4:**
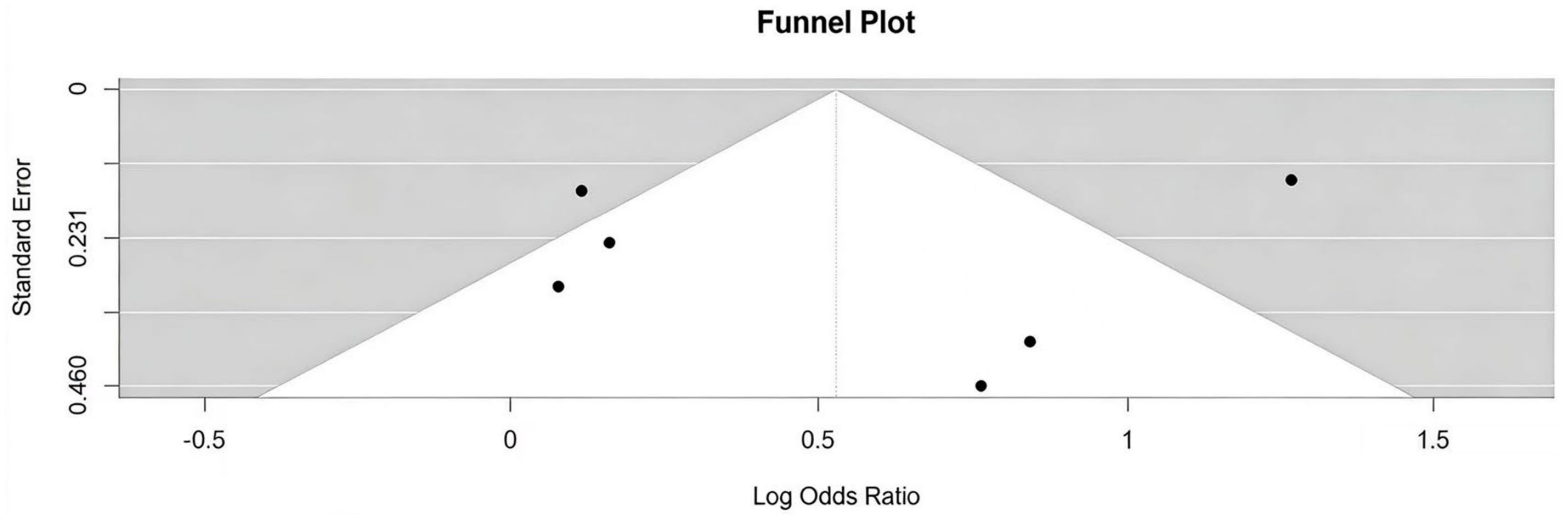
Funnel plot of standard error by log odds ratio. Funnel plot of standard error by log odds ratio. Based on the six studies included in the final meta-analysis. Begg’s test (*p* = 0.719) and Egger’s test (*p* = 0.996) indicated no significant publication bias.

Publication bias was assessed through funnel plots constructed using log-transformed odds ratios (log ORs), supplemented by Begg’s and Egger’s statistical tests (Figure 4). Visual inspection of the funnel plot showed a symmetrical distribution, indicating no apparent publication bias. This observation was supported by quantitative analysis. Specifically, Begg’s test yielded Kendall’s *τ* = 0.20 (*p* = 0.719), and Egger’s test produced an intercept of 0.01 (*p* = 0.996). Both tests confirmed the absence of a significant correlation between effect sizes and standard errors, demonstrating no statistical evidence of publication bias.

## 4 Discussion

This study is the first to systematically confirm an association between frailty and depression in patients with CHD through meta-analysis, addressing a critical knowledge gap in cardiovascular comorbidity research. While this association has been documented in general older population (24), its characterization in CHD patients has been limited by methodological inconsistencies and insufficient sample sizes in prior investigations.

This finding aligns with and extends previous research in cardiovascular populations. For instance, a recent study by Rawashdeh et al. reported similar associations in post-myocardial infarction patients (19), while Huang et al. found that frailty predicted depressive symptoms in a cohort of heart failure patients (30). The consistency of these findings across different cardiovascular conditions suggests that the frailty-depression link may be a common feature of chronic heart disease.

This association is likely underpinned by shared biological mechanisms. Chronic inflammation, for instance, has been implicated in both conditions. Elevated levels of interleukin-6 (IL-6) and other pro-inflammatory cytokines can promote atherosclerosis, accelerate muscle catabolism that contributes to frailty, and impair dopaminergic neurotransmission, which may lead to depressive symptoms (31–33). Dysregulation of the hypothalamic pituitary adrenal (HPA) axis results in prolonged cortisol elevation. This disrupts endothelial function and causes hippocampal damage, thereby exacerbating both frailty and depression (26, 34–36). Mitochondrial dysfunction and oxidative stress cause imbalances in energy metabolism, which jointly contribute to physical decline and mood disturbances (37, 38). These mechanisms collectively suggest a complex pathophysiological interplay between frailty and depression in CHD, which may contribute to self-perpetuating clinical deterioration cycles.

Our meta-analysis found a pooled OR of 2.45 (95% CI: 1.22–4.92) for the association between frailty and depression in CHD patients. By comparison, a previous meta-analysis in the general older population reported a pooled OR of 1.62 (95% CI: 1.28–2.04) (39). While direct comparison across different meta-analyses should be interpreted cautiously due to differences in study populations, designs, and measurement tools, the larger effect size observed in our study raises the possibility of CHD-specific factors that may amplify this association. Psychologically, the diagnosis of CHD introduces unique stressors, including disease uncertainty and fear of recurrence (19). These factors promote fatigue development, leading to concurrent neuropsychological impairment and physical functional decline (20).

Sensitivity analysis confirmed that excluding one low-quality study (NOS score ≤ 5) did not alter the direction or significance of the pooled estimate, supporting the robustness of our findings. Substantial heterogeneity persisted (*I^2^* = 90%) even after this exclusion, indicating that variability across studies is driven by multiple factors beyond a single outlier.

Subgroup analyses provided some insights into potential sources of this heterogeneity. Differences by study design suggested that cohort studies yielded larger pooled effect size (OR = 3.92; 95% CI: 1.66–9.27) than cross-sectional studies (OR = 1.91; 95% CI: 1.02–3.58). This difference may reflect the ability of longitudinal designs to better capture temporal patterns of the association, potentially by reducing recall bias and allowing more precise ascertainment of exposure and outcome. However, the small number of cohort studies (*n* = 2) and residual heterogeneity within this subgroup (*I^2^* = 71%) warrant caution in interpretation. Nevertheless, the consistency of a positive association across both designs strengthens confidence in the overall finding.

Differences were also observed by assessment tool and geographic region. Studies using TFI demonstrated significantly higher associations (OR = 6.75, 95% CI: 2.79–16.36) than those using CFS (OR = 2.24, 95% CI: 0.91–5.56), potentially reflecting TFI’s psychological dimension weighting. Geographically, European populations showed higher effect sizes (OR = 6.75) than Asian counterparts (OR = 1.42), suggesting cultural norms influence somatic symptom reporting. These findings underscore the need for standardized assessment approaches in future research.

Beyond these explored factors, several other sources of heterogeneity likely contribute to the observed variability. These include differences in study populations (e.g., age distribution, CHD severity, comorbidity burden), variations in the definition and measurement of CHD itself, and inconsistent adjustment for confounders across studies. Additionally, unmeasured factors such as medication use, socioeconomic status, and social support may influence both frailty and depression and could contribute to between-study variability. The high residual heterogeneity underscores the complexity of the association between frailty and depression in CHD patients and highlights the need for future studies with standardized methodologies and comprehensive reporting of patient characteristics.

It is important to note that several subgroup comparisons should be interpreted with caution. The estimates for some assessment tools (e.g., PHQ-2, CFS, CGA) and the European and American geographic subgroups were each derived from single studies. Findings based on single studies are particularly susceptible to the influence of individual study characteristics. Therefore, these observations should be considered preliminary and need confirmation in future research with larger numbers of studies per subgroup.

Several limitations should be considered when interpreting our findings. First, although our search strategy did not restrict inclusion by age, all eligible studies were conducted in older adults (aged ≥60 years). This uniformity reflects the current state of the literature, where research on frailty in CHD has predominantly focused on older populations, likely due to the age-dependent nature of frailty itself. While this enhances the internal consistency of our pooled estimate, it limits generalizability to younger patients with CHD, a population of growing clinical importance. The association in younger patients may differ due to variations in disease etiology, psychosocial context, and physiological reserve, warranting future research in this group.

Second, causal inference is constrained by the predominance of cross-sectional studies (4/6). Although all included studies met quality thresholds (AHRQ score ≥ 6, NOS ≥ 6), key confounders such as CHD severity and comorbidity burden were not consistently adjusted for. Additionally, other potential sources of heterogeneity, including variations in sample age, specific comorbidities (e.g., diabetes, hypertension), and medication use, were inconsistently reported. Prospective longitudinal designs with comprehensive covariate control are needed to address these gaps.

Despite these limitations, our findings have clinical implications. The significant association between frailty and depression in older CHD patients supports the potential value of incorporating frailty screening into routine care for those presenting with depressive symptoms, and vice versa. Given the complex interplay between these conditions, multimodal interventions (e.g., integrated exercise and psychological support programs) may offer benefits. Looking forward, four research priorities emerge: (1) developing and validating standardized frailty and depression assessment tools specific to CHD populations to improve diagnostic comparability (40). (2) Conducting well-designed longitudinal cohort studies to clarify temporal patterns and inform intervention timing (41). (3) Evaluating the risk–benefit profiles of antidepressants in CHD patients, addressing current uncertainties about cardiovascular safety (42). (4) Testing collaborative care models that integrate multidisciplinary approaches for comprehensive symptom management (43, 44).

## 5 Limitations and future directions

Although neither the funnel plot nor Egger’s test indicated evidence of publication bias, the small number of included studies (*n* = 6) may have limited the statistical power of these assessments. Moreover, the exclusion of non-English literature potentially introduced geographic and language biases.

The predominance of cross-sectional studies (67%) substantially limits causal inference regarding the association between frailty and depression in CHD patients. Future research should increase the use of longitudinal designs to better capture the temporal patterns of this association. In addition, the short follow-up durations in existing cohort studies limit the ability to evaluate long-term trajectories. Therefore, future research should include extended follow-up periods to more comprehensively assess the evolving association between frailty and depression.

Another important limitation concerns the handling of effect estimates. The majority of pooled estimates in this meta-analysis were derived from crude (unadjusted) odds ratios, as most included studies did not report adjusted estimates accounting for potential confounders. While crude ORs provide a useful measure of the overall association, they do not account for variables that may influence both frailty and depression, such as age, sex, comorbidities (e.g., diabetes, hypertension, heart failure), CHD severity, and medication use. The absence of adjustment for these factors means that the observed association may be influenced by residual confounding. Future studies should report fully adjusted models to enable more precise estimation of the independent association between frailty and depression in CHD patients.

The small number of included studies (*n* = 6) also limited the precision of our pooled estimates and reduced the statistical power of subgroup analyses. Although we conducted pre-specified subgroup analyses, some subgroups contained only one study, making these findings exploratory rather than definitive. Substantial heterogeneity in the measurement of both frailty and depression across studies, including different assessment tools, cut-off values, and time frames, may have contributed to the observed variability. This measurement heterogeneity limits direct comparability between studies and underscores the urgent need for standardized assessment protocols in future research.

Furthermore, the choice of odds ratio as the effect measure itself warrants consideration. Both frailty and depression have relatively high prevalence in CHD populations, with reported rates often exceeding 20–50%. When outcomes are common (prevalence >10%), odds ratios may overestimate the magnitude of association compared with risk ratios or prevalence ratios. This issue is particularly relevant for the cross-sectional studies that constitute the majority of our included studies. Although risk ratios would theoretically be more appropriate, they were not reported in the primary studies and could not be calculated from the available data. Therefore, our pooled OR estimates should be interpreted with this potential overestimation in mind. Future studies should report risk ratios or provide sufficient data to allow calculation of alternative effect measures.

Finally, as discussed in detail above, all included studies focused on older adults with CHD (aged ≥60 years). While this reflects the current evidence base and enhances internal consistency, it limits the generalizability of our findings to younger patients with CHD, a population of growing clinical importance. The implications of this and directions for future research are further elaborated in the Discussion section.

## 6 Conclusion

In conclusion, this meta-analysis found a significant association between frailty and depression in older patients with CHD, based on the currently available evidence. The consistency of this finding across different study designs and populations supports its robustness, although the high heterogeneity and predominance of cross-sectional studies warrant cautious interpretation. Future well-designed longitudinal studies with standardized assessments are needed to clarify the temporal nature of this association and to inform integrated management strategies for this high-risk population.

## Data Availability

The original contributions presented in the study are included in the article/Supplementary material, further inquiries can be directed to the corresponding author.
