# Supplementary material for: Association between frailty and depression in older adults with coronary heart disease: a systematic review and meta-analysis
Source: Front Public Health. 2026 Apr 13;14:1737823. doi: 10.3389/fpubh.2026.1737823 (PMC13110990; doi:10.3389/fpubh.2026.1737823)
Supplement: Supplementary file 1 [file Supplementary_file_1.docx]

**Table 1. The search and screening strategy.**

| Database | Search strategy | Items found |
| --- | --- | --- |
| PubMed | #1 "heart"[MeSH Terms] OR "Atherosclerosis"[MeSH Terms] OR "angina pectoris"[MeSH Terms] OR "coronary disease*"[Title/Abstract] OR "disease* coronary"[Title/Abstract] OR "coronary heart disease*"[Title/Abstract] OR "disease* coronary heart"[Title/Abstract] OR "heart disease* coronary"[Title/Abstract] OR "coronary artery disease*"[Title/Abstract] OR "artery disease* coronary"[Title/Abstract] OR "coronary arteriosclerosis"[Title/Abstract] OR "Arteriosclerosis"[Title/Abstract] OR "coronary atherosclerosis"[Title/Abstract] OR "arteriosclerosis coronary"[Title/Abstract] OR "myocardial infarction*"[Title/Abstract] OR "infarction* myocardial"[Title/Abstract] OR "cardiovascular stroke*"[Title/Abstract] OR "stroke* cardiovascular"[Title/Abstract] OR "myocardial infarct*"[Title/Abstract] OR "infarct* myocardial"[Title/Abstract] OR "heart attack*"[Title/Abstract] OR "angina pectoris"[Title/Abstract] OR "Angina"[Title/Abstract] OR "Stenocardia"[Title/Abstract] OR "acute coronary syndrome*"[Title/Abstract] OR "coronary syndrome* acute"[Title/Abstract] OR "syndrome* acute coronary"[Title/Abstract] | 1,051,744 |
|  | #2 "Frailty"[MeSH Terms] OR "Frailties"[Title/Abstract] OR "Frailness"[Title/Abstract] OR "frailty syndrome"[Title/Abstract] OR "Debility"[Title/Abstract] OR "Debilities"[Title/Abstract] OR "frail"[Title/Abstract] | 30,254 |
|  | #3 "depressive disorder"[MeSH Terms] OR "depression"[MeSH Terms] OR "depress*"[Title/Abstract] OR "depression"[Title/Abstract] OR "depressive disorder"[Title/Abstract] | 672,231 |
|  | #4 #1 AND #2 AND #3 | 44 |
| Web of Science | #5 TS=(heart OR Atherosclerosis OR angina pectoris OR coronary disease* OR disease* coronary OR coronary heart disease* OR disease* coronary heart OR heart disease* coronary OR coronary artery disease* OR artery disease* coronary OR coronary arteriosclerosis OR Arteriosclerosis OR coronary atherosclerosis OR arteriosclerosis coronary OR myocardial infarction* OR infarction* myocardial OR cardiovascular stroke* OR stroke* cardiovascular OR myocardial infarct* OR infarct* myocardial OR heart attack* OR angina pectoris OR Angina OR Stenocardia OR acute coronary syndrome* OR coronary syndrome* acute OR syndrome* acute coronary) | 3,691,818 |
|  | #6 TS=(Frailty OR Frailties OR Frailness OR frailty syndrome OR Debility OR Debilities OR frail) | 75,072 |
|  | #7 TS=(depressive disorder OR depression OR depress* OR depression OR depressive disorder) | 1,279,331 |
|  | #8 #5 AND #6 AND #7 | 754 |
| The Cochrane Library | #9 MeSH descriptor: [heart] explode all trees OR MeSH descriptor: [Atherosclerosis] explode all trees OR MeSH descriptor: [angina pectoris] explode all trees OR (coronary disease*): ti, ab, kw OR (disease* coronary): ti, ab, kw OR (coronary heart disease*): ti, ab, kw OR (disease* coronary heart): ti, ab, kw OR (heart disease* coronary): ti, ab, kw OR (coronary artery disease*): ti, ab, kw OR (artery disease* coronary): ti, ab, kw OR (coronary arteriosclerosis): ti, ab, kw OR (Arteriosclerosis): ti, ab, kw OR (coronary atherosclerosis): ti, ab, kw OR (arteriosclerosis coronary): ti, ab, kw OR (myocardial infarction*): ti, ab, kw OR (infarction* myocardial): ti, ab, kw OR (cardiovascular stroke*): ti, ab, kw OR (stroke* cardiovascular): ti, ab, kw OR (myocardial infarct*): ti, ab, kw OR (infarct* myocardial): ti, ab, kw OR (heart attack*): ti, ab, kw OR (angina pectoris): ti, ab, kw OR (Angina): ti, ab, kw OR (Stenocardia): ti, ab, kw OR (acute coronary syndrome*): ti, ab, kw OR (coronary syndrome* acute): ti, ab, kw OR (syndrome* acute coronary): ti, ab,kw | 95,963 |
|  | #10 MeSH descriptor: [Frailty] explode all trees OR (Frailties): ti, ab, kw OR (Frailness): ti, ab, kw OR (frailty syndrome): ti, ab, kw OR (Debility): ti, ab, kw OR (Debilities): ti, ab, kw OR (frail): ti, ab, kw | 4,529 |
|  | #11 MeSH descriptor: [depressive disorder] explode all trees OR MeSH descriptor: [depression] explode all trees OR (depress*): ti, ab, kw OR (depression): ti, ab, kw OR (depressive disorder): ti, ab, kw | 123,430 |
|  | #12 #9 AND #10 AND #11 | 15 |
| Embase | #13 'heart'/exp OR 'angina pectoris'/exp OR 'coronary disease*': ti, ab, kw OR 'disease* coronary': ti, ab, kw OR 'coronary heart disease*': ti, ab, kw OR 'disease* coronary heart': ti, ab, kw OR 'heart disease* coronary': ti, ab, kw OR 'coronary artery disease*': ti, ab, kw OR 'artery disease* coronary': ti, ab, kw OR 'coronary arteriosclerosis': ti, ab, kw OR 'arteriosclerosis': ti, ab, kw OR 'coronary atherosclerosis': ti, ab, kw OR 'arteriosclerosis coronary': ti, ab, kw OR 'myocardial infarction*': ti, ab, kw OR 'infarction* myocardial': ti, ab, kw OR 'cardiovascular stroke*': ti, ab, kw OR 'stroke* cardiovascular': ti, ab, kw OR 'myocardial infarct*': ti, ab, kw OR 'infarct* myocardial': ti, ab, kw OR 'heart attack*': ti, ab, kw OR 'angina pectoris': ti, ab, kw OR 'angina': ti, ab, kw OR 'stenocardia': ti, ab, kw OR 'acute coronary syndrome*': ti, ab, kw OR 'coronary syndrome* acute': ti, ab, kw OR 'syndrome* acute coronary': ti, ab, kw | 1,555,316 |
|  | #14 'frailty'/exp OR 'frailties': ti, ab, kw OR 'frailness': ti, ab, kw OR 'frailty syndrome': ti, ab, kw OR 'debility': ti, ab, kw OR 'debilities': ti, ab, kw OR 'frail': ti, ab, kw |  |
|  | #15 'depression'/exp OR 'depress*': ti, ab, kw OR 'depression': ti, ab, kw OR 'depressive disorder': ti, ab, kw | 1,088,072 |
|  | #16 #13 AND #14 AND #15 | 187 |
| CINAHL | #17 (MM"heart") OR (MM"Atherosclerosis") OR (MM"angina pectoris") OR TI (coronary disease* OR disease* coronary OR coronary heart disease* OR disease* coronary heart OR heart disease* coronary OR coronary artery disease* OR artery disease* coronary OR coronary arteriosclerosis OR Arteriosclerosis OR coronary atherosclerosis OR arteriosclerosis coronary OR myocardial infarction* OR infarction* myocardial OR cardiovascular stroke* OR stroke* cardiovascular OR myocardial infarct* OR infarct* myocardial OR heart attack* OR angina pectoris OR Angina OR Stenocardia OR acute coronary syndrome* OR coronary syndrome* acute OR syndrome* acute coronary) OR AB (coronary disease* OR disease* coronary OR coronary heart disease* OR disease* coronary heart OR heart disease* coronary OR coronary artery disease* OR artery disease* coronary OR coronary arteriosclerosis OR Arteriosclerosis OR coronary atherosclerosis OR arteriosclerosis coronary OR myocardial infarction* OR infarction* myocardial OR cardiovascular stroke* OR stroke* cardiovascular OR myocardial infarct* OR infarct* myocardial OR heart attack* OR angina pectoris OR Angina OR Stenocardia OR acute coronary syndrome* OR coronary syndrome* acute OR syndrome* acute coronary) | 104,842 |
|  | #18 MM"Frailty" OR TI (Frailties OR Frailness OR frailty syndrome OR Debility OR Debilities OR frail) OR AB (Frailties OR Frailness OR frailty syndrome OR Debility OR Debilities OR frail) | 18,945 |
|  | #19 (MM"depressive disorder") OR (MM"depression") OR TI (depress* OR depression OR depressive disorder) OR AB (depress* OR depression OR depressive disorder) | 285,066 |
|  | #20 #17 AND #18 AND #19 | 9 |
| Scopus | #21 TITLE-ABS-KEY ("heart" OR "Atherosclerosis" OR "angina pectoris" OR "coronary disease*" OR "disease* coronary" OR "coronary heart disease*" OR "disease* coronary heart" OR "heart disease* coronary" OR "coronary artery disease*" OR "artery disease* coronary" OR "coronary arteriosclerosis" OR "Arteriosclerosis" OR "coronary atherosclerosis" OR "arteriosclerosis coronary" OR "myocardial infarction*" OR "infarction* myocardial" OR "cardiovascular stroke*" OR "stroke* cardiovascular" OR "myocardial infarct*" OR "infarct* myocardial" OR "heart attack*" OR "angina pectoris" OR "Angina" OR "Stenocardia" OR "acute coronary syndrome*" OR "coronary syndrome* acute" OR "syndrome* acute coronary") | 3,251,875 |
|  | #22 TITLE-ABS-KEY ("Frailty" OR "Frailties" OR "Frailness" OR "frailty syndrome" OR "Debility" OR "Debilities" OR "frail") | 67,065 |
|  | #23 TITLE-ABS-KEY ("depressive disorder" OR "depression" OR "depress*" OR "depression" OR "depressive disorder") | 1,190,347 |
|  | #24 #21 AND #22 AND #23 | 1,151 |
| Grey database | GreyNet International: http://greynet.org  OPENGREY: http://www.opengrey.eu  NTIS: <http://www.ntis.gov>  APA: PsycExtra http://www.apa.org/psycextra/ | 0 |
